# Metagenomic analysis of endemic viruses in oral secretions from Chinese pigs

**DOI:** 10.1002/vms3.869

**Published:** 2022-09-01

**Authors:** Sajid Umar, Benjamin D. Anderson, Kuanfu Chen, Guo‐Lin Wang, Mai‐Juan Ma, Gregory C. Gray

**Affiliations:** ^1^ Global Health Research Center Duke Kunshan University Kunshan Jiangsu China; ^2^ Division of Natural and Applied Sciences Duke Kunshan University Kunshan Jiangsu China; ^3^ State Key Laboratory of Pathogen and Biosecurity Beijing Institute of Microbiology and Epidemiology Beijing China; ^4^ Division of Infectious Diseases University of Texas Galveston USA; ^5^ Program in Emerging Infectious Diseases Duke‐NUS Medical School Singapore

**Keywords:** China, coinfection, detection, ecology, phylogeny, pigs, virus

## Abstract

**Background:**

Pigs are unique reservoirs for virus ecology. Despite the increased use of improved biosecurity measures, pig viruses readily circulate in Chinese swine farms.

**Objectives:**

The main objective of this study was to examine archived swine oral secretion samples with a panel of pan‐species viral assays such that we might better describe the viral ecology of swine endemic viruses in Chinese farms.

**Methodology:**

Two hundred (*n* = 200) swine oral secretion samples, collected during 2015 and 2016 from healthy pigs on six swine farms in two provinces in China, were screened with molecular pan‐species assays for coronaviruses (CoVs), adenoviruses (AdVs), enteroviruses (EVs), and paramyxoviruses (PMV). Samples were also screened for porcine circovirus (PCV) 3, porcine reproductive and respiratory syndrome virus (PRRSV) and influenza A virus (IAV).

**Results:**

Among 200 swine oral secretion samples, 152 (76.0%) were found to have at least one viral detection. Thirty‐four samples (17%) were positive for more than one virus, including 24 (70.5%) with dual detection and 10 (29.5%) with triple detection. Seventy‐eight (39.0%) samples were positive for porcine AdVs, 22 (11.0%) were positive for porcine CoVs, 21 (10.5%) were positive for IAVs, 13 (6.5%) were positive for PCV, 7 (3.5%) were positive for PMV, six (3.0%) were positive for PRRSV and five (2.5%) were positive for porcine EV.

**Conclusion:**

Our findings underscore the high prevalence of numerous viruses among production pigs in China and highlight the need for routine, periodic surveillance for novel virus emergence with the goal of protecting pigs.

## Graphical abstract

Pigs are unique animal reservoirs for pathogen transmission. Coinfections are more frequent in pig farms than single infections.

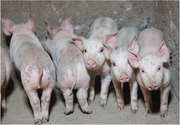

## 1 INTRODUCTION

Pigs are considered important reservoirs for multiple pathogens and provide unique environments for virus ecology. In the past decade, the world has experienced the emergence and spread of a number of novel pig viruses that occasionally spill over to humans. The rapid expansion of the commercial swine industry has contributed to the emergence and rapid spread of swine viruses that could be a major threat to the pig industry worldwide, including China. A high prevalence of swine viruses is found in large herds, and new variants are routinely being discovered (Hause & Scheidt 2016; Ramesh et al., 2021). Recently, a review article (VanderWaal & Deen, 2018) summarised publications during the last 50 years and identified pigs as hosts harboring many viruses. Mounting evidence suggests that coinfections are more prevalent in modern swine farms than single pathogen infections (Anderson et al., 2018; Gray & Baker, 2011; Guo et al., 2020; Ma et al., 2015; Ma et al., 2018; Saade et al., 2020; Sun et al., 2015). Although several studies have explored swine virus ecology, such studies have seldom examined virus ecology in Chinese swine farms (VanderWaal & Deen, 2018), and fewer still have examined viral coinfections among Chinese pigs.

Among swine pathogens, a variety of infectious agents are shed in oral fluid, including many of the most economically important. Oral fluid sampling is a non‐invasive and simple method to study swine pathogens at the herd level (Prickett et al., 2008). In this study, we examined archived swine oral secretion samples collected for influenza A virus (IAV) surveillance (Anderson et al 2018) with a panel of pan‐species viral assays such that we might better describe the viral ecology of swine endemic viruses in Chinese farms.

## 2 MATERIALS AND METHODS

### 2.1 Study design

This study was approved by the institutional review boards of Duke University and the Beijing Institute of Microbiology and Epidemiology. During March 2015, as part of an ongoing 5‐year prospective epidemiological study to assess IAV ecology, six Chinese swine farms (three each in Jiangsu and Shandong Provinces) were visited on a monthly basis to collect samples from pigs at different stages of production (growers, finishers, sows and boars). The enrolled farms varied in size (0.6–4 km^2^), the average number of pigs on‐site per day (310–2500) and the number of swine houses (3–27) (Anderson et al., 2018; Ma et al., 2018). Briefly, data captured for each specimen included the location, age and gender of the pigs in each pen (Table 1). Pig oral secretion (POS) samples were collected using a hanging rope method where three‐strand braided unbleached 100% cotton ropes, with 5/8″ diameter, were pre‐soaked with a 5% sterile glucose solution and placed in swine pens (Anderson et al., 2018; Ma et al., 2018). Ropes were attached to a rod or pole and placed 40 cm above the ground for 20 to 30 mins during which time the pigs would chew on the rope. At the conclusion of the sampling, oral fluids were manually and aseptically expressed from the rope into a sterile sampling bag (Cat. No. EPR‐4590, Labplas, Inc.). Samples were transported under cold chain methods to Chinese public health laboratories where processing and initial IAV molecular assays were conducted.

**TABLE 1:** Molecular results of swine oral secretion samples collected from six Chinese swine farms in 2015

						Infection status						
Farm number	Collection date	Sample number	Pig type	Pig age (weeks)	Site number	CoV	AdV	PMV	EV‐G	PRRSV	PCV3	IAV
SF02	1 July 2015	POS0962	Production pig	10	28‐N4	–	+	–	–	–	+	+
SF02	1 July 2015	POS0964	Production pig	10	28‐S2	–	+	–	–	–	+	+
SF02	1 July 2015	POS0965	Production pig	10	28‐N5	–	+	–	–	–	–	+
SF02	1 July 2015	POS0967	Production pig	10	28‐N11	–	+	–	–	–	–	+
SF02	1 July 2015	POS0971	Production pig	10	28‐S6	–	+	–	+	–	+	–
SF02	1 July 2015	POS0977	Boar	104	23‐S2	–	+	–	–	–	+	–
SF02	1 July 2015	POS0986	Production pig	16	21‐S1	–	–	–	–	–	+	–
SF02	1 July 2015	POS0987	Production pig	16	21‐S2	–	–	–	–	–	–	–
SF02	1 July 2015	POS0991	Production pig	8	27‐S2	–	+	–	–	–	–	–
SF02	1 July 2015	POS0992	Production pig	8	27‐S3	–	+	–	–	–	–	–
SF02	1 July 2015	POS0993	Production pig	8	27‐N2	+	+	–	–	–	+	–
SF02	1 July 2015	POS0995	Production pig	8	27‐S4	–	–	–	–	–	–	–
SF02	1 July 2015	POS0999	Production pig	8	27‐S6	–	+	–	–	–	–	–
SF03	6 July 2015	POS1007	Production pig	20	1‐1	–	+	–	–	–	–	–
SF03	6 July 2015	POS1009	Production pig	20	1‐2	–	+	–	–	–	+	–
SF03	6 July 2015	POS1010	Production pig	16	1‐7	–	+	–	–	–	–	–
SF03	6 July 2015	POS1012	Production pig	16	1‐5	–	–	–	–	–	–	+
SF03	6 July 2015	POS1013	Production pig	16	1‐4	–	+	–	–	–	–	–
SF03	6 July 2015	POS1014	Production pig	16	1‐4	–	+	–	–	–	–	–
SF03	6 July 2015	POS1016	Boar	76	4E‐N3	–	–	–	–	–	+	–
SF03	6 July 2015	POS1018	Boar	104	4E‐S2	–	+	–	–	–	–	–
SF03	6 July 2015	POS1022	Production pig	8	3‐S5	–	+	–	–	–	–	–
SF03	6 July 2015	POS1024	Production pig	10	3‐S7	–	+	–	–	–	–	+
SF03	6 July 2015	POS1026	Production pig	10	3‐S9	–	+	–	–	–	+	–
SF03	6 July 2015	POS1029	Production pig	16	1‐5	–	+	–	–	–	–	–
SF03	6 July 2015	POS1030	Production pig	16	1‐6	+	+	–	+	–	–	–
SF03	6 July 2015	POS1032	Production pig	16	1‐8	–	+	–	+	–	–	–
SF03	6 July 2015	POS1033	Boar	156	4E‐N6	–	+	–	–	–	–	–
SF03	6 July 2015	POS1037	Production pig	12	1‐12	–	+	–	–	–	–	–
SF03	6 July 2015	POS1039	Production pig	6	4W‐S1	–	+	–	+	–	–	+
SF03	6 July 2015	POS1041	Production pig	6	4W‐N3	+	+	–	–	–	–	+
SF03	6 July 2015	POS1046	Production pig	6	4W‐S4	–	+	–	+	–	–	+
SF03	6 July 2015	POS1048	Production pig	6	4W‐N7	–	+	–	–	–	–	–
WF04	17 July 2015	6 July 2015	Production pig	4	1	–	+	–	–	–	–	–
WF04	17 July 2015	POS1068	Production pig	4	1	–	+	–	–	–	–	–
WF04	17 July 2015	POS1070	Production pig	4	1	–	+	–	–	–	+	–
WF04	17 July 2015	POS1071	Production pig	24	3	–	–	–	–	–	+	–
WF04	17 July 2015	POS1072	Production pig	24	3	–	+	–	–	–	–	–
WF04	17 July 2015	POS1076	Production pig	24	3	–	+	–	–	–	–	–
WF04	17 July 2015	POS1077	Production pig	24	3	–	+	–	–	–	–	–
WF04	17 July 2015	POS1080	Production pig	24	3	–	+	–	–	–	–	–
WF04	17 July 2015	POS1089	Production pig	24	3	–	+	–	–	–	–	–
WF04	17 July 2015	POS1090	Production pig	24	3	–	–	–	–	–	–	–
WF04	17 July 2015	POS1094	Production pig	16	14	–	–	–	–	+	–	–
WF04	17 July 2015	POS1096	Production pig	16	14	–	–	–	–	+	–	–
WF04	17 July 2015	POS1098	Production pig	16	14	–	–	–	–	–	+	–
WF04	17 July 2015	POS1100	Production pig	16	14	–	+	–	–	–	–	–
WF05	16 July 2015	POS1103	Sow	130	PSP3	–	+	–	–	–	+	–
WF05	16 July 2015	POS1104	Sow	130	PSP3	–	–	–	–	–	–	–
WF05	16 July 2015	POS1106	Sow	130	PSP3	–	+	–	–	–	–	–
WF05	16 July 2015	POS1107	Sow	130	PSP3	–	–	–	–	–	–	–
WF05	16 July 2015	POS1110	Sow	130	PSP3	–	+	–	–	–	–	–
WF05	16 July 2015	POS1113	Production pig	9	NP2	–	–	–	–	–	–	–
WF05	16 July 2015	POS1115	Production pig	9	NP2	–	–	–	–	–	–	–
WF05	16 July 2015	POS1116	Production pig	9	NP2	+	–	–	–	–	–	–
WF05	16 July 2015	POS1118	Production pig	9	NP2	–	–	–	–	–	–	–
WF05	16 July 2015	POS1121	Production pig	9	NP2	–	–	–	–	+	–	+
WF05	16 July 2015	POS1130	Production pig	9	NP2	–	–	–	–	–	–	–
WF05	16 July 2015	POS1131	Production pig	12	PPP3	–	+	–	–	–	–	–
WF05	16 July 2015	POS1132	Production pig	12	PPP3	–	+	–	–	–	–	–
WF05	16 July 2015	POS1135	Production pig	12	PPP3	+	+	–	–	–	–	–
WF05	16 July 2015	POS1145	Production pig	24	PPP4	–	–	–	–	+	–	–
WF05	16 July 2015	POS1149	Production pig	24	PPP4	–	–	–	–	–	–	–
WF06	17 July 2015	POS1152	Production pig	20	PPP2	–	–	–	–	+	–	–
WF06	17 July 2015	POS1153	Production pig	20	PPP2	–	–	–	–	–	–	–
WF06	17 July 2015	POS1155	Production pig	20	PPP2	–	–	–	–	–	–	–
WF06	17 July 2015	POS1157	Production pig	20	PPP2	–	+	–	–	–	–	–
WF06	17 July 2015	POS1164	Production pig	20	PPP2	+	+	–	–	–	–	–
WF06	17 July 2015	POS1165	Production pig	20	PPP2	+	+	–	–	–	–	–
WF06	17 July 2015	POS1166	Production pig	20	PPP2	+	+	–	–	–	–	–
WF06	17 July 2015	POS1169	Production pig	20	PPP2	+	+	–	–	–	–	–
WF06	17 July 2015	POS1170	Production pig	20	PPP2	+	+	–	–	–	–	+
WF06	17 July 2015	POS1173	Production pig	24	PPP1	+	–	–	–	–	–	–
WF06	17 July 2015	POS1177	Production pig	24	PPP1	+	–	–	–	–	–	+
WF06	17 July 2015	POS1178	Production pig	24	PPP1	+	+	+		–	–	–
WF06	17 July 2015	POS1181	Production pig	16	PPP5	–	+	–	–	–	–	–
WF06	17 July 2015	POS1192	Production pig	16	PPP5	+	+	–	–	–	–	–
WF06	17 July 2015	POS1194	Production pig	16	PPP5	–	–	–	–	–	–	–
SF01	31 July 2015	POS1202	Production pig	20	S2‐1	–	–	–	–	–	–	–
SF01	31 July 2015	POS1206	Production pig	8	S2‐7	–	+	–	–	–	–	–
SF01	31 July 2015	POS1207	Production pig	20	S2‐3	–	–	+	–	–	–	–
SF01	31 July 2015	POS1208	Production pig	16	S2‐4	–	–	–	–	–	–	–
SF01	31 July 2015	POS1212	Production pig	12	S2‐10	–	+	–	–	–	–	–
SF01	31 July 2015	POS1214	Production pig	8	S2‐11	–	+	–	–	–	–	–
SF01	31 July 2015	POS1221	Production pig	12	S2‐10	+	+	–	–	–	–	–
SF01	31 July 2015	POS1222	Production pig	8	S2‐7	+	–	–	–	–	–	–
SF01	31 July 2015	POS1224	Production pig	20	S2‐3	–	–	–	–	–	–	–
SF01	31 July 2015	POS1225	Production pig	16	S2‐4	+	–	–	–	–	–	–
SF01	31 July 2015	POS1230	Production pig	20	S2‐1	+	–	–	–	–	–	–
SF01	31 July 2015	POS1239	Production pig	8	S2‐11	–	+	–	–	–	–	–
SF01	31 July 2015	POS1242	Sow	104	S1‐7	+	–	–	–	–	–	–
SF02	30 July 2015	POS1257	Production pig	20	28‐EN2	–	+	–	–	–	–	–
SF02	30 July 2015	POS1259	Sow	104	18‐S7	–	–	–	–	–	–	–
SF02	30 July 2015	POS1261	Production pig	20	28‐WS4	–	+	–	–	–	–	–
SF02	30 July 2015	POS1262	Production pig	20	28‐ES3	–	–	–	–	–	–	–
SF02	30 July 2015	POS1263	Production pig	20	28‐ES4	–	+	–	–	–	–	–
SF02	30 July 2015	POS1265	Production pig	20	28‐EN4	–	–	–	–	–	–	–
SF02	30 July 2015	POS1276	Boar	104	23‐N2	+	–	–	–	–	–	–
SF02	30 July 2015	POS1282	Production pig	24	21‐ES2	–	+	–	–	–	–	–
SF02	30 July 2015	POS1283	Production pig	24	21‐EN3	–	+	+	–	–	–	–
SF02	30 July 2015	POS1286	Production pig	24	21‐EN9	–	–	–	–	–	–	–
SF02	30 July 2015	POS1287	Production pig	24	21‐S8	–	–	–	–	–	–	–
SF02	30 July 2015	POS1289	Production pig	24	21‐S10	–	–	–	–	–	–	–
SF02	30 July 2015	POS1290	Production pig	24	21‐S11	+	–	–	–	–	–	–
SF03	31 July 2015	POS1301	Sow	104	2‐N1	–	–	–	–	+	–	–
SF03	31 July 2015	POS1306	Production pig	8	3‐S4	–	+	–	–	–	–	–
SF03	31 July 2015	POS1307	Production pig	8	3‐S3	–	+	+		−	–	–
SF03	31 July 2015	POS1311	Production pig	14	1‐7	–	+	–	–	–	–	–
SF03	31 July 2015	POS1321	Production pig	16	1‐10	–	+	–	–	–	–	–
SF03	31 July 2015	POS1330	Production pig	20	1‐4	–	+	–	–	–	–	–
SF03	31 July 2015	POS1331	Production pig	20	1‐5	–	+	–	–	–	–	–
SF03	31 July 2015	POS1339	Production pig	6	4W‐N1	–	+	–	–	–	–	–
SF03	31 July 2015	POS1344	Production pig	6	4W‐S5	–	+	+	–	–	–	–
WF04	20 August 2015	POS1351	Production pig	4	1	–	–	+	–	–	–	+
WF04	20 August 2015	POS1352	Production pig	4	1	–	–	–	–	–	–	+
WF04	20 August 2015	POS1359	Production pig	4	1	–	+	–	–	–	–	–
WF04	20 August 2015	POS1360	Production pig	4	1	–	+	–	–	–	–	+
WF04	20 August 2015	POS1361	Production pig	4	1	–	–	–	–	–	–	+
WF04	20 August 2015	POS1366	Production pig	4	1	–	+	–	–	–	–	–
WF04	20 August 2015	POS1372	Production pig	24	2	–	–	+	–	–	–	–
WF04	20 August 2015	POS1389	Production pig	20	15	–	+	–	–	–	–	+
WF04	20 August 2015	POS1390	Production pig	20	15	–	+	–	–	–	–	–
WF04	20 August 2015	POS1395	Production pig	20	15	+	­	–	–	–	–	–
WF05	20 August 2015	POS1438	Production pig	20	PPP3	–	+	–	–	–	–	–
WF06	20 August 2015	POS1457	Production pig	28	PPP2	–	–	–	–	–	–	–
WF06	20 August 2015	POS1465	Production pig	28	PPP2	–	–	–	–	–	–	–
WF06	20 August 2015	POS1475	Production pig	12	PPP3	–	+	–	–	–	–	–
WF06	20 August 2015	POS1476	Production pig	12	PPP3	–	+	–	–	–	–	–
WF06	20 August 2015	POS1492	Production pig	20	PPP5	–	+	–	–	–	–	–
WF06	20 August 2015	POS1496	Production pig	20	PPP5	–	+	–	–	–	–	–

Abbreviations: AdV, adenovirus; CoV, coronavirus; EV‐G, enterovirus species G; IAV, influenza A virus; PCV3, porcine circovirus‐3; PMV, paramyxovirus; POS, pig oral secretion; PRRSV, porcine reproductive and respiratory syndrome virus.

### 2.2 Sample selection and laboratory analyses

At Duke Kunshan University, a total of 200 samples were selected from a database of 2700 original samples using random number generating software (https://www.random.org/). Simultaneous viral DNA and RNA extraction were performed using QIAamp MinElute Virus Spin Kits (Qiagen) following the manufacturer's recommendations. Positive and negative controls were used during each extraction to validate the extraction procedure and reagent integrity. The total genomic extraction of each sample was assessed using gel‐based PCR assays with the Platinum Taq DNA Polymerase Kit (Thermo Fisher Scientific, Inc.) for the detection of pan‐species adenovirus (AdV; Gray et al., 2021) and porcine circovirus 3 (PCV3); Palinski et al., 2017). The viral RNA of each sample was assessed with gel‐based Reverse transcriptase‐polymerase chain reaction (RT‐PCR) assays using the SuperScript III Platinum One‐Step RT‐PCR System with Platinum Taq DNA Polymerase (Thermo Fisher Scientific, Inc.) for the detection of pan‐species enterovirus (EV), pan‐species coronavirus (CoV), pan‐species paramyxovirus (Gray et al., 2021; Xiu et al., 2020) and porcine reproductive and respiratory syndrome virus (PRRSV; Xie et al., 2013). In addition, the viral RNA of each sample was screened for IAV by a qRT‐PCR assay targeting the influenza matrix genome segment using a one‐step RT‐PCR kit (Cat. No. 56046, TaKaRa) on an Applied Biosystems 7500 real‐time PCR platform (Life Technologies) as previously described (Ma et al., 2018). All PCR runs had a negative template control (nuclease‐free water) and a corresponding synthetic positive control sample included.

### 2.3 Sequencing and phylogenetic analyses

Partial genome sequencing of positive samples was performed by a commercial sequencing company (Genewiz). Assembly and analysis of sequence data were conducted using BioEdit Software version 5.0.9. This program was also used to edit the sequencing electropherograms and to exclude nucleotide ambiguity. Multiple sequence alignments were performed using ClustalW (Thompson et al., 1994). Sequences were submitted to national center for biotechnology information (NCBI) GenBank under the following accession numbers CoV: MZ271775‐MZ271786, AdV: MZ271787‐MZ271793, EV species G (EV‐G): MZ271794‐MZ271798, PCV3: MZ271799‐MZ271806 and PRRSV: MZ271807–MZ271808. To understand the molecular epidemiology of identified viruses in this study, closely related sequences (based on identity score) from viruses in GenBank were downloaded to infer the overall detected virus phylogeny. The NCBI's basic local alignment search tool application and BioEdit 7.1.9 (Ibis Biosciences) were employed. Sequences were aligned using the neighbour‐joining method in MEGA X (Kumar et al., 2018). A bootstrap analysis was performed to assess the confidence limits of the branching with 1000 replicates.

## 3 RESULTS

Overall, 152 (76.0%) of the 200 POS samples had molecular evidence of at least one virus. Seventy‐eight (39.0%) samples were positive for porcine AdV, 22 (11.0%) were positive for porcine CoV, 21 (10.5%) were positive for IAV, 13 (6.5%) were positive for PCV3, 7 (3.5%) were positive for paramyxovirus (PMV), 6 (3.0%) were positive for PRRSV and 5 (2.5%) samples were positive for porcine EV‐G. Multiple virus coinfections were inferred. Twenty‐four samples had evidence of dual infection, and 10 samples had evidence of triple infection (Tables 1 and 2). Seven samples had detection of both CoV and AdVs, followed by five with IAV + AdV and AdV + PCV3, three with AdV + PMV and one each with AdV + EV‐G, IAV + PMV, swine influenza virus (SIV) + PRRSV and IAV + CoV. Of the 10 samples with triple infection, two samples each were positive for AdV + PCV3 + IAV, AdV + CoV + IAV or AdV + EV‐G + IAV, and one sample each was positive for AdV + CoV + PMV, AdV + EV‐G + PCV3, AdV + CoV + EV‐G or AdV + CoV + PCV3.

**TABLE 2:** Summary of coinfection status for molecularly tested pig oral secretion (POS) samples collected in 2015

**Infection status**	**Virus**	**Infection status 200 POS samples**		
Single infection		**Positive number**	**Percentage**	**Total percentage**
	IAV	7	3.5%	37%
	CoV	9	4.5%	
	AdV	47	23.5%	
	EV‐G	0	0%	
	PRRSV	5	2.5%	
	PCV3	4	2%	
	PMV	2	1%	
Dual infection	AdV + CoV	7	3.5%	12%
	AdV + PCV3	5	2.5%	
	AdV + IAV	5	2.5%	
	AdV + EV‐G	1	0.5%	
	AdV + PMV	3	1.5%	
	IAV + PMV	1	0.5%	
	SIV + PRRSV	1	0.5%	
	IAV + CoV	1	0.5%	
Triple infection	AdV + CoV+ PMV	1	0.5%	5%
	AdV + EV‐G + PCV3	1	0.5%	
	AdV + PCV3 + IAV	2	1%	
	AdV + CoV + EV‐G	1	0.5%	
	AdV + CoV + IAV	2	1%	
	AdV + CoV + PCV3	1	0.5%	
	AdV + EV‐G + IAV	2	1%	
Totally	IAV	21	10.5%	76%
	CoV	22	11%	
	AdV	78	39%
	EV‐G	5	2.5%	
	PRRSV	6	3%	
	PCV3	13	6.5%	
	PMV	7	3.5%	

Abbreviations: AdV, adenovirus; CoV, coronavirus; EV‐G, enterovirus species G; IAV, influenza A virus; PCV3, porcine circovirus‐3; PMV, paramyxovirus; PRRSV, porcine reproductive and respiratory syndrome virus; SIV, swine influenza virus.

**FIGURE 1:**
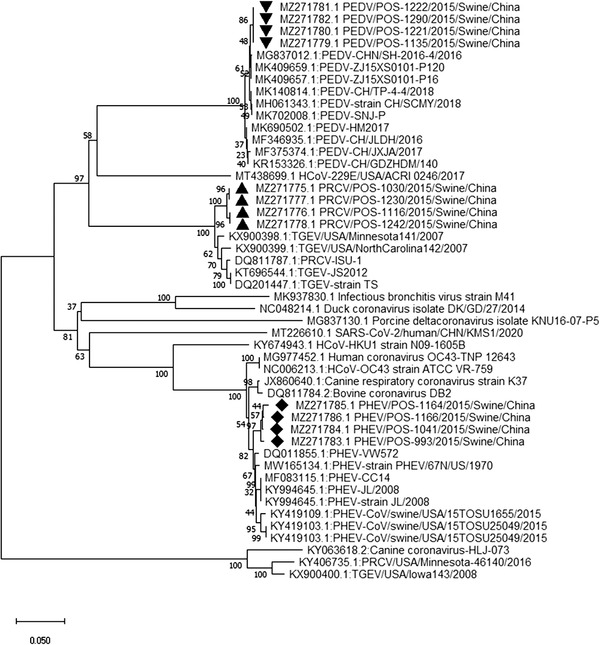
Phylogenetic analysis based on RNA‐dependent RNA polymerase of coronavirus (CoV) strains using the neighbour‐joining tree method and p‐distance model using MEGA version 10 (http://www.megasoftware.net). Bootstrap values were calculated on 1000 replicates

**FIGURE 2:**
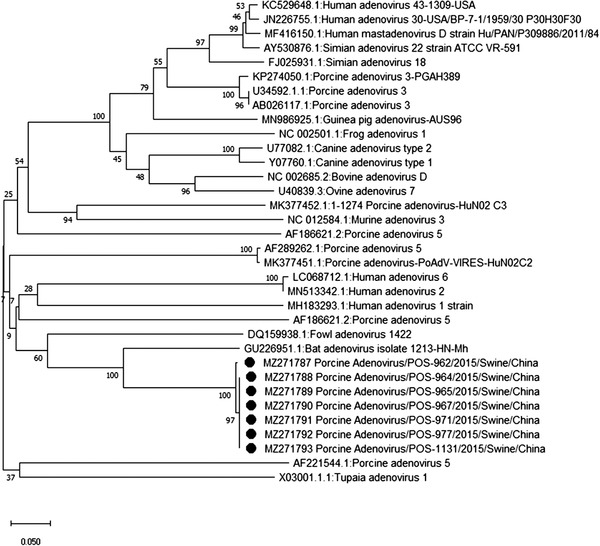
Phylogenetic analysis based on DNA polymerase of HAdV strains using the neighbour‐joining tree method and p‐distance model using MEGA version 10 (http://www.megasoftware.net). Bootstrap values were calculated on 1000 replicates

**FIGURE 3:**
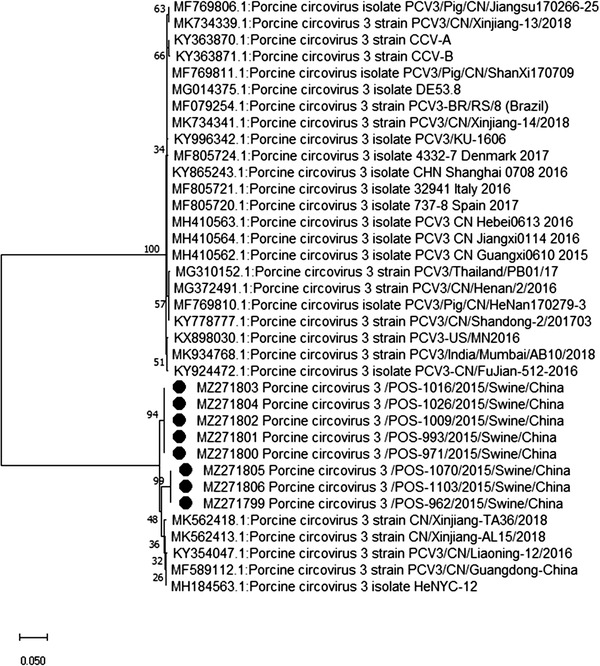
Phylogenetic analysis based on the capsid gene of porcine circovirus 3 (PCV3) strains using the neighbour‐joining tree method and p‐distance model using MEGA version 10 (http://www.megasoftware.net). Bootstrap values were calculated on 1000 replicates

**FIGURE 4:**
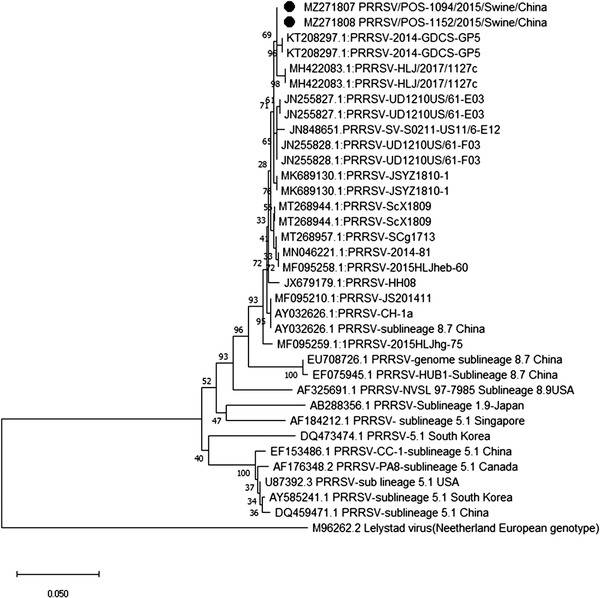
Phylogenetic analysis based on the *ORF5* gene of porcine reproductive and respiratory syndrome virus (PRRSV) strains using the neighbour‐joining tree method and p‐distance model using MEGA version 10 (http://www.megasoftware.net). Bootstrap values were calculated on 1000 replicates

**FIGURE 5:**
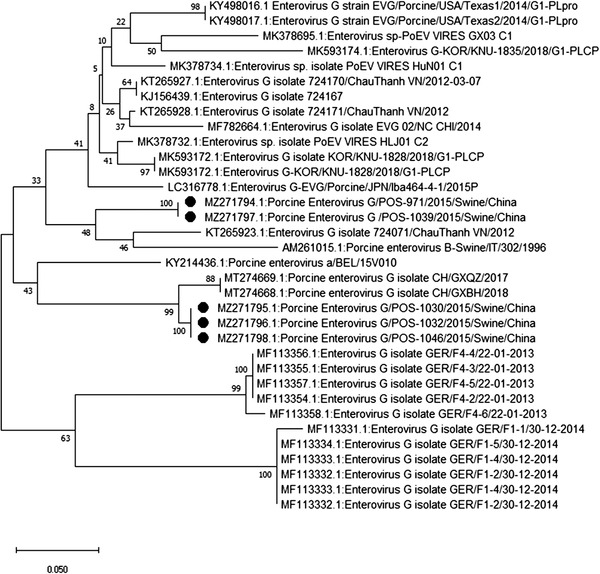
Phylogenetic analysis based on the *VP1* gene of enterovirus species G (EV‐G) strains using the neighbour‐joining tree method and p‐distance model using MEGA version 10 (http://www.megasoftware.net). Bootstrap values were calculated on 1000 replicates

Sequencing and phylogenetic analysis of specimens positive for CoV (MZ271775‐MZ271786), AdV (MZ271787‐MZ271793), EV‐G (MZ271794‐MZ271798), PCV3 (MZ271799‐MZ271806) and PRRSV (MZ271807 ‐ MZ271808) revealed a close association with porcine CoV, porcine AdV, porcine EV‐G, PCV3 and PRRSV (Figures 1, 2, 3, 4, 5), all of which have been previously reported in China. Unfortunately, positive specimens for paramyxovirus did not yield successful sequences. Various types of porcine CoV, including porcine haemagglutinating encephalomyelitis virus (14/22), porcine respiratory CoV (4/22) and porcine epidemic diarrhoea virus (4/22), were detected in our study. The sequencing results for IAV were previously published (Ma et al., 2018) showing the presence of swine‐lineage H1N1, H3N2 and A(H1N1)pdm09‐like viruses in the study farms.

## 4 DISCUSSION

Our findings are consistent with the high prevalence of swine pathogens found in the limited other related studies conducted in China. Chen et al. (2019) reported singular infections (32.7%), dual infections (15.72%) and multiple infections (3.1%) caused by CSFV, PRRSV, PCV and PCV3. Other studies have shown that PRRSV and PCV2 coinfection rates are 21.9%–52.3% (Ge et al., 2012; Liu et al., 2013). The detected prevalence of PCV2 and PCV3 coinfection ranged from 6.8% to 39.4% among swine samples (Zhang et al., 2018). The prevalence of CSFV and PRRSV coinfection varied from 0% to 7.7% in different regions of China (Liu et al., 2011). PRRSV and PCV3 coinfection in China has also been previously detected (Chen et al., 2017). Furthermore, CSFV, PRRSV and PCV2 coinfection was also observed in previous studies, with prevalence rates ranging from 2.5% to 3.6% (Liu et al., 2013; Xu et al., 2012). A more recent study demonstrated that 12.9%, 36.0% and 1.8% of PRRSV‐positive pigs were coinfected with PCV2, PRRSV and CSF, respectively (Zhou et al., 2020).

We also performed phylogenetic analysis of sequence data to understand the diversity of each virus. The identity of the nucleotide sequence of porcine AdV (PAdV serotype 5, species C), compared with previously reported sequences from China, varied from 96.9% to 97.2%. The percent identity of the porcine CoV nucleotide sequence varied from 97.3% to 99.4%. The percent identity of the PCV3 (genotype PCV3b) nucleotide sequence varied from 98.9% to 99.2%. The alignment of the sequences among the PRRSV strains in this study showed 99.6% to 100% nucleotide similarity for the *ORF5* gene. Alignment of the sequences among the EV strains revealed EV‐G (86%–96%). EV‐G is prevalent and widespread in the general pig population in middle and eastern China, and infections tend to occur early, usually within the first week after birth (Mi et al., 2021).

None of the PMV‐positive samples was successfully sequenced. Therefore, PMV genotyping was unable to be assessed. Sequencing results revealed the detection of swine‐lineage H1N1 and H3N2 and A(H1N1) pdm09‐like viruses, which were closely related to viruses previously identified in China. The re‐assortment between H1N1pdm09 and SIVs has drawn attention, as coinfection of pigs with SIV and avian/human‐source influenza strains can contribute to the evolution of new influenza viruses with pandemic potential for humans (Ding et al., 2021). Similarly, all other viruses (PCV3, EV, PRRSV, AdV) in the current study showed close association with previously reported viruses from China, indicating that these viruses have now become endemic and continuously circulate in Chinese swine farms. Our phylogenetic analyses are consistent with previously published reports from China (Chen et al., 2017; Chen et al., 2019; Jiang et al., 2020; Zhang et al., 2018; Zheng et al., 2020).

This study had several limitations. We did not test for PCV2 or African swine fever virus and lacked case comparisons for pathogenicity. We also did not assess the seasonality of swine viruses. In addition, virus isolation for this study was not attempted, making it difficult to know if our molecular detections represented viable viruses. In convenient sampling, we assumed that the pigs that did not chew the rope were not sampled and considered for the analysis. Samples were archived for further characterisation, including infectivity experiments. Additionally, positive specimens for PMV could not yield sequencing data, possibly due to the low concentration of RNA in the samples.

In conclusion, this study supports the notion that pigs in China are often coinfected with multiple viruses, a number of which are known to have pathogenic potential to pigs. This study identified different patterns of coinfection along with singular infection. In addition, phylogenetic analyses suggested that the detected viruses were enzootic in multiple herds at different locations in China. Overall, these data reinforce the premise that viral pathogens are highly prevalent among China's swine herds. These findings highlight the need for routine, periodic surveillance for novel virus emergence in Chinese swine farms with the goal of protecting swine herds.

## CONFLICTS OF INTEREST

The authors declare that they have no conflicts of interest regarding the publication of this article.

## AUTHOR CONTRIBUTIONS

*Formal analysis, methodology, writing–original draft*: Sajid Umar. *Conceptualisation, data curation, investigation, supervision, writing–review and editing*: Benjamin D. Anderson. *Formal analysis, investigation, methodology*: Kuanfu Chen.*Conceptualisation, data curation, validation*: Guo‐Lin Wang. *Data curation, investigation, project administration, resources*: Mai‐Juan Ma. *Conceptualisation, funding acquisition, project administration,resources, validation, writing–review and editing*: Gregory C. Gray.

## FUNDING INFORMATION

NIH/NIAID grant R01AI108993‐01A1; the National Natural Science Foundation of China (Grant Numbers: 81402730 and 81773494; the Beijing Science and Technology Nova Program (Grant Number: Z171100001117088) and the Program of International Science and Technology Cooperation of China (Grant Number: 2013DFA30800)

## ETHICS STATEMENT

This study was approved by the institutional review boards of Duke University (Pro00056116), Duke Kunshan University and the Academy of Military Medical Sciences. Institutional Animal Care and Use Committee approvals were also granted by Duke University (A187‐14‐08) and the Academy of Military Medical Sciences (AMMS‐20‐14‐009).

### PEER REVIEW

The peer review history for this article is available at https://publons.com/publon/10.1002/vms3.869.

## Data Availability

None.
